# Adriamycin-induced podocyte injury via the Sema3A/TRPC5/Rac1 pathway

**DOI:** 10.3389/fmed.2024.1381479

**Published:** 2024-09-05

**Authors:** Yan Liu, Ri-Li Ge, Zhen-Zhen Shan, Yan-Jun Wang, Yan-Yan Yang, Xue Sun, Peng-Li Luo

**Affiliations:** ^1^Key Laboratory of High Altitude Medicine (Ministry of Education), Key Laboratory of Application and Foundation for High Altitude Medicine Research in Qinghai Province (Qinghai-Utah Joint Research Key Lab for High Altitude Medicine), Research Center for High Altitude Medicine, Qinghai University, Xining, China; ^2^Clinical Research Center for Chronic Kidney Disease in Qinghai Province, Xining, China

**Keywords:** podocytopathy, Sema3A, podocyte, TRPC5, foot process effacement

## Abstract

Podocytopathies encompass kidney diseases where direct or indirect podocyte injury leads to proteinuria or nephrotic syndrome. Although Semaphorin3A (Sema3A) is expressed in podocytes and tubular cells in adult mammalian kidneys and has a common effect on the progression of podocyte injury, its mechanism remains unclear. Previous studies have shown increased Sema3A expression in various glomerulopathies, indicating a gap in understanding its role. In this study, analysis of human data revealed a positive correlation between the levels of urinary Sema3A and Podocalyxin (PCX), suggesting a close relationship between Sema3A and podocyte loss. Furthermore, the impact of Adriamycin on podocytes was investigated. Adriamycin induced podocyte migration and apoptosis, along with an increase in Sema3A expression, all of which were ameliorated by the inhibition of Sema3A. Importantly, TRPC5 was found to increase the overexpression of Sema3A in podocytes. A TRPC5 inhibitor, AC1903, alleviated podocyte migration and apoptosis, inhibiting the formation of lamellar pseudopodia in the podocyte cytoskeleton by lowering the expression of Rac1. Furthermore, AC1903 relieved massive albuminuria and foot process effacement in the kidneys of Adriamycin-treated mice *in vivo*. In conclusion, our findings suggest that Sema3A may impact the cytoskeletal stability of podocytes through TRPC5 ion channels, mediated by Rac1, ultimately leading to foot process effacement. Notably, AC1903 demonstrates the potential to reverse Adriamycin-induced foot process fusion and urine protein. These results contribute to a deeper understanding of the mechanisms involved in podocytopathies and highlight the therapeutic potential of targeting the Sema3A-TRPC5 pathway.

## 1 Introduction

The global estimated prevalence of chronic kidney disease (CKD) is 13.4%, with end-stage kidney disease (ESKD) requiring renal replacement therapy estimated between 4.902 million and 7.083 million (1, 2). CKD is associated with a high risk of early disability and necessitates high-cost treatments, such as hemodialysis, peritoneal dialysis, and kidney transplantation in cases of end-stage renal failure (3, 4). The majority of kidney diseases progressing to CKD initiate in the glomeruli, with glomerulopathy significantly accelerating podocyte loss (5). Halting podocyte injury may, thus, protect patients from glomerulosclerosis progression.

The glomerular filtration barrier, comprising podocytes, basement membranes, and glomerular endothelial cells, is integral for normal kidney function. Podocytopathies, are a group of proteinuria glomerular disorders or nephrotic syndrome driven by direct or indirect podocyte injury (6). Proteinuria is identified as an independent risk factor for progression to ESKD (7).

Semaphorins, a family of secretory or membrane-binding glycoproteins, exert control over cell migration and axon growth cone guidance (8). Previous studies have associated Semaphorin3Awith renal diseases (9, 10). While typically undetectable in human urine, Sema3A becomes detectable in acute renal injury caused by hypoxia, ischemia, and nephrotoxic drugs (11). It is considered an early predictive biomarker for experimental kidney disease and AKI (12). Reports indicate elevated urinary Sema3A levels in patients with minimal change nephropathy (MCD), membranous nephropathy (MN), and IgA nephropathy (IgAN) compared to controls (13). In both diabetic and non-diabetic patients with hypertension, urinary Sema3A is independently associated with CKD (14). These findings suggest a common impact of Seam3A on podocyte injury and renal damage progression in humans. Animal research demonstrates occluding junctions joining podocyte foot processes in Sema3A−/− mice kidneys, with widened foot processes compared to wild-type mice (15). Sema3A induces transient proteinuria via podocyte foot process effacement and fusion (16). However, the mechanism of Sema3A action in foot process effacement (FPE) remains unclear.

In this study, we investigated the role of Sema3A and TRPC5 in an Adriamycin-induced podocyte model, utilizing the small-molecule TRPC5 ion channel inhibitor, AC1903 (17), both *in vivo* and *in vitro*, to examine its therapeutic effect.

## 2 Materials and methods

### 2.1 Materials

Adriamycin was purchased from Aladdin Biochemical Technology Co., Ltd. (Shanghai, China), while AC1903 was obtained from Selleck Chem (Tokyo, Japan). Recombinant mouse Semaphorin3A protein from Sino Biological (Beijing, China). Antibodies against Sema3A, Podocin, and Rac1 were purchased from Sigma-Aldrich. TRPC5, Desmin, and Sema3A assay kits were sourced from Abcam, Millipore Biological Engineering Co., Ltd. (Wuhan, China), and Shanghai Fusheng Industrial Co., Ltd. (Shanghai, China), respectively.

### 2.2 Human study

The study encompassed 114 Chinese patients aged 18–79 years who underwent renal biopsy and 21 thyroid nodules patients for control at Qinghai University Affiliated Hospital between 2020 and 2022. All patients were diagnosed based on renal biopsy pathology and clinical manifestations. All methods were performed in accordance with the relevant guidelines and regulations. This study was approved by the Ethics Review Committee of the Affiliated Hospital of Qinghai University (approval number: P-SL-2023-473).

### 2.3 Cell culture and treatment

Mouse podocytes were provided by Beijing Longyue Biotechnology Development Co. Ltd. The podocytes were cultured in RPMI 1640 medium containing 10% fetal bovine serum and 10 U/mL mouse recombinant interferon-γ at 33°C. To induce differentiation, cells were Thermoshifted to 37°C and incubated in an interferon-free medium for 14 days. Upon reaching 70–80% confluency, podocytes were exposed to Adriamycin at a final concentration of 0.25 μg/mL for 24 h. The final concentrations of Sema3A and AC1903 were 100 μM and 4.05 μM, respectively. The cells were divided into eight groups.

### 2.4 Transfection with small interfering RNA

Podocytes (1 × 10^5^ cells/mL) were seeded into a 6-well plate (2 mL/well) and transfected with Sema3A mall interfering RNA (siRNA) using Exfect2000 Transfection Reagent. siRNA served as a transfection control. Six hours post-infection, the medium was replaced with a fresh complete culture medium. Cells were harvested 24 h later, and the expression level of Sema3A was examined by western blotting to evaluate infection efficiency.

### 2.5 Construction of EGFP-N1 plasmid containing Sema3A cDNA

mRNA was extracted from the podocyte cell line, and total cDNA was obtained using a reverse transcription kit (cat.). The mouse Sema3A cDNA sequence was acquired from NCBI, and primers were designed with EcoRI and AgeI restriction sites at both ends. The Sema3A cDNA fragment containing the restriction site was cloned using the total cDNA as a template. The resulting fragment, along with the EGFP-N1 plasmid, was digested overnight with restriction enzymes targeting EcoRI (Cat.). The fragment was then linked to T4 DNA ligase (cat. no. 1). The resulting fragment was an EGFP-N1 plasmid containing Sema3A cDNA, which was transferred to DH5α, and monoclones were screened using agarose-LB plates containing kanamycin. After monoclonal amplification, the plasmid was extracted and sequenced to confirm the presence of Sema3A cDNA.

### 2.6 Selection of plasmid transfection and Sema3A overexpression monoclonal cell lines

The EGFP-N1 plasmid containing the Sema3AcDNA fragment and EGFP-N1 empty plasmid were transfected into the cell lines. Observations were made for 48 h, and cells were continuously passaged. After a week, the digested cells were diluted to 100 cells/10 mL/100 μL/well, added to 96-well plates, and incubated. Four weeks later, when the cells were full, green fluorescent cells containing only one monoclonal cell mass were labeled and transferred to 6-well plates for further culture. After reaching cell confluency, proteins were obtained from digested cells, and the expression of Sema3AcDNA in transfected and untransfected cells was detected using qPCR.

### 2.7 Cell viability assay

Podocytes were cultured in 96-well plates at a density of 2,000 cells/well and subjected to different treatments. The OD values were detected at a wavelength of 450 nm using a Multiskan MK3 microplate reader.

### 2.8 Cell migration assay

Cells (5 × 10^5^ cells/well) were plated in a 6-well plate and incubated until they reached confluence. The monolayer was scratched using a pipette tip and washed with a serum-free medium to remove detached cells. Subsequently, cells were cultured in a complete medium supplemented with or without AN (100 μL/well) and siRNA-Sema3A. Podocytes were photographed at 0 and 24 h after wounding, and the closure area of the wound was calculated using the formula: migration area (%) = (A_0_ – A_24_)/A_0_ × 100, where A_n_ represents the remaining wound area at n h.

### 2.9 Transwell invasion assay

Cells (2 × 10^5^ cells/well) were suspended in low serum medium and seeded into the upper chamber of 24-well Transwell plates with 8 μm pore filters. Then, the lower chamber was filled with a complete medium supplemented with or without AN (100 μL/well) and siRNA-Sema3A. After 12 h, cells attached to the upper surface of the filter membranes were removed, and the migrated cells on the lower surface were stained with 0.5% crystal violet for 15–30 min. The migration level was observed under an optical microscope (Leica DMI6000B, Wetzlar, Germany).

### 2.10 Annexin V-FITC/propidium iodide staining for detecting cell apoptosis

The cells were collected at specific time points, washed once with cold PBS, and centrifuged (1,500 rpm, 5 min). The microspheres were suspended in a 500 μL binding buffer containing Annexin V-FITC and propidium iodide (PI) solution and stained in the dark for 5 min at 20°C-25°C. The percentage of apoptotic cells was determined using a BD Accuri C6 Human Flow Cytometer (Piscataway, NJ, United States).

### 2.11 Western blotting

Podocytes were homogenized with RIPA buffer at 4°C for 30 min and centrifuged at 12,000 rpm for 10 min to obtain cell supernatant. After boiling and denaturation, samples were separated using 10% sodium dodecyl sulfate-polyacrylamide gel electrophoresis (SDS-PAGE) at 80 V and 100 V for 30 and 90 min, respectively. Electrophoresis was halted when bromophenol blue reached approximately 0.5 cm from the bottom of the gel. The gel was removed and balanced in a transfer buffer for 20 min; the sample was transferred onto a polyvinylidene fluoride (PVDF) film (250 mA, 120 min). The membranes were blocked with 5% skim milk powder. Primary antibodies were incubated at a ratio of 1:1,000 or 1:2,000 on a shaking bed (overnight at 4°C) and washed with TPBS four times. Secondary antibodies (diluted 1:5,000) were incubated at room temperature for 2 h. The cells were washed with TPBS seven times. After continuous exposure to the X-ray film, the immune response bands were quantitatively analyzed using ImageJ software. Relative expression was calculated according to the ratio of each band expression to that of the internal reference GAPDH.

### 2.12 Fluorescence confocal microscopy

Podocytes were placed in a confocal dish, incubated for 24 h, washed three times with PBS, fixed with 4% paraformaldehyde, permeabilized, and finally blocked with 0.3% Triton X-100 and 10% goat serum. F-actin was stained with Alexa-phalloidin. After three washes with PBS, nuclei were stained with 4′,6-diamino-2-phenylindole (DAPI). Images were obtained using a confocal laser scanning microscope (Zeiss, Germany).

### 2.13 Animals

Male BALB/c mice, weighing 20–25 g, were provided with SPF mouse maintenance feed (Chengdu Dashuo Laboratory Animal Co., Ltd.) and injected with a single dose of Adriamycin (AN) (10 mg/kg) into the posterior bulbar sinus. After AN injection, drug loading or AC1903 (50 mg/kg) was administered twice daily (8 a.m. and 8 p.m.) for 10 days, and metabolic specimens were collected on the 10th day before euthanasia (1% Pentobarbital Sodium, 50 mg/kg, Intraperitoneal injection). Two kidneys were collected for downstream experiments: one was used for liquid nitrogen flash freezing, and the other was fixed in advance with 3% glutaraldehyde or overnight with 4% PFA and stored in PBS for subsequent experiments. Nineteen mice were studied (Control, AN, AN + AC1903; *n* = 5, 7, 7, respectively). The AC1903 solution was placed on a heated shaker at 48°C and 800 rpm, with 2 mL of drug/compound per kilogram of body weight. Weighing was measured at the time of injection.

### 2.14 Transmission electron microscopy

The samples were fixed with 3% glutaraldehyde and 1% osmium tetroxide, dehydrated with acetone in series, impregnated with epoxy resin 812 for a longer time, and then embedded. Semi-thin sections were stained with methylene blue, and ultrathin sections were cut with diamond knives, uranyl acetate, and lead citrate. The sections were examined using a JEM-1400-FLASH transmission electron microscope.

### 2.15 Statistical analysis

Data were expressed as mean ± standard deviation ( $\bar{x}\pm s)$ . Pearson’s or Spearman’s correlation coefficients were used to determine the correlations between two variables. The differences between the two groups were analyzed using a two-tailed Student’s *t*-test. ANOVA or Welch’s test was used for comparisons among multiple groups, with the Bonferroni or Games–Howell post-test. Analyses were performed using SPSS 28.0 (Inc, IL, United States) and GraphPad Prism TM 5.0 (San Diego, CA, United States). The difference between groups was considered statistically significant at *p* < 0.05.

## 3 Results

### 3.1 Correlation between urinary Sema3A levels, urinary PCX, and 24-h urinary protein levels in patients with podocytopathies

**Figure 1:**
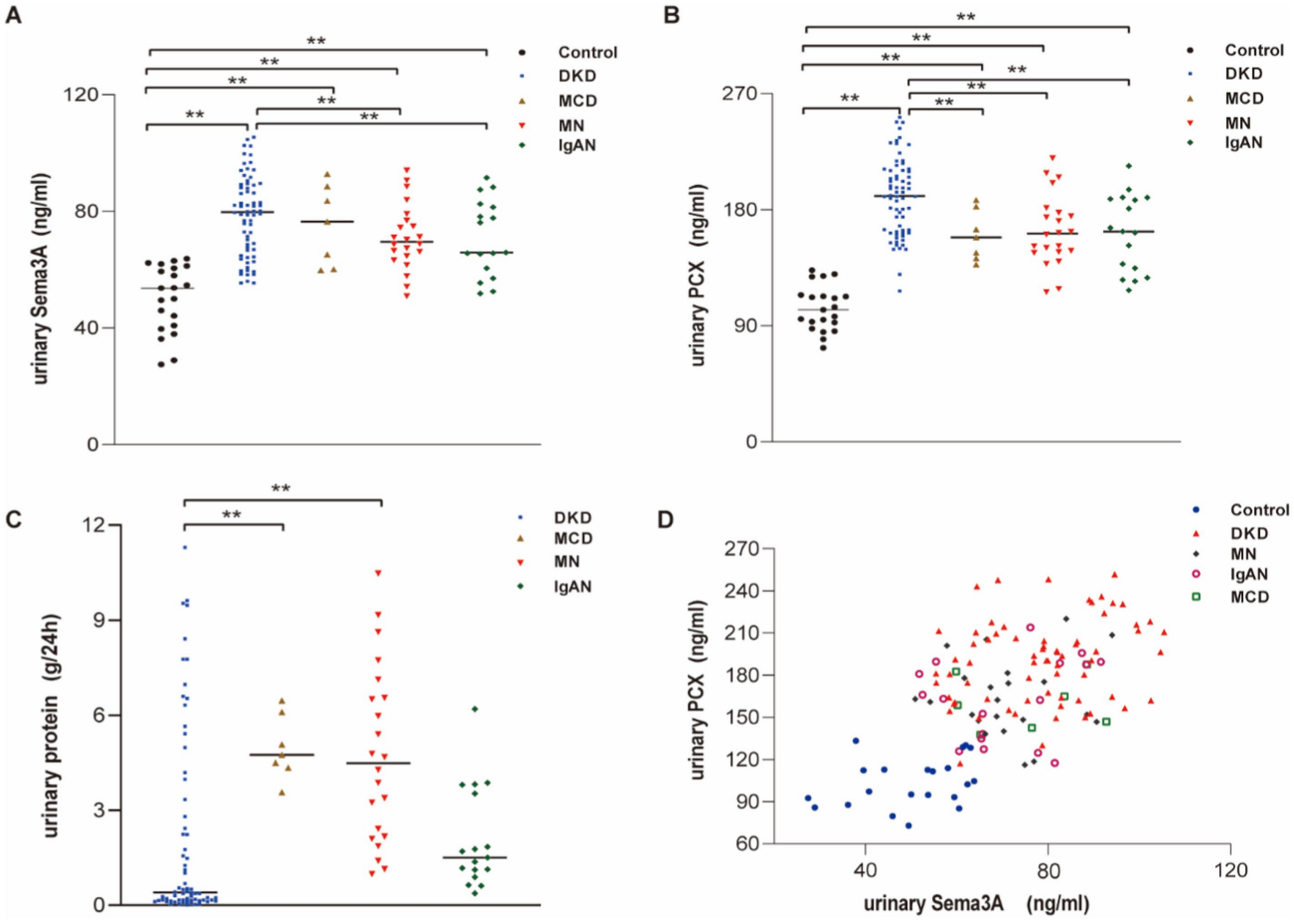
Urinary Sema3A and 24 h urinary protein levels in patients with podocytopathies and controls. **(A)** Urinary Sema3A in patients with podocytopathies and controls. ^**^p < 0.01. **(B)** Urinary PCX in patients with podocytopathies and controls. ^**^p < 0.01. **(C)** Twenty-four-hour urinary protein in patients with podocytopathies. ^**^p < 0.01. **(D)** Scatter plot between urinary Sema3A and PCX (*n* = 135).

To investigate the relationship between podocytopathy and Sema3A, we initially assessed urinary Sema3A and urinary PCX levels in patients with various podocytopathies. The characteristics of the patient groups are shown in Supplementary Table S1. Statistical analysis revealed elevated levels of urinary Sema3A (Figure 1A) and PCX (Figure 1B) in patients with podocytopathy compared to those in controls. Additionally, 24-h urinary protein levels in MCD and MN were higher than those in the control group (Figure 1C). Urinary Sema3A level exhibited a positive correlation with urinary podocyte PCX in 114 patients with podocytopathies and 21 controls (*r* = 0.566, *p* < 0.001) (Figure 1D). As shown in Table 1, a non-significant correlation was observed between urinary Sema3A and 24-h urinary protein levels in patients with podocytopathy, while a positive correlation was observed between urinary PCX and 24-h urinary protein levels in patients with Diabetic kidney disease (DKD).

**Table 1:** Correlation of urinary protein (g/24 h) levels with urinary Sema3A (ng/ml) and PCX (ng/ml) levels in podocytopathies patients.

	Sema3A (ng/ml)				PCX (ng/ml)			
	DKD	MN	IgAN	MCD	DKD	MN	IgAN	MCD
*n*	68	22	17	7	68	22	17	7
*r_s_*	0.068	0.014	−0.294	0.607	0.464	0.330	−0.130	−0.214
*p*	0.583	0.950	0.252	0.148	<0.001	0.133	0.619	0.645

### 3.2 Adriamycin-induced podocyte migration and apoptosis and reduced cell viability

**Figure 2:**
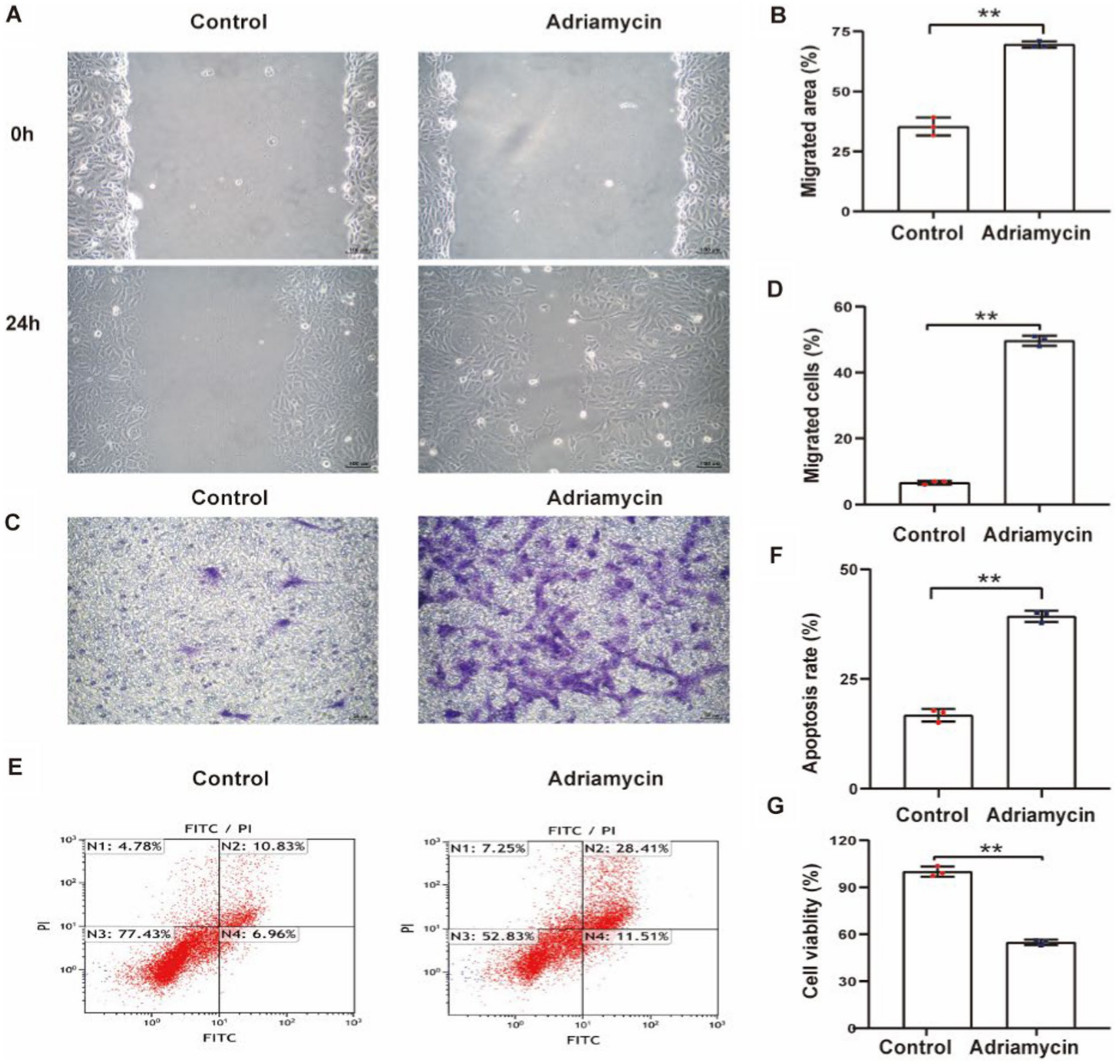
Adriamycin inhibits podocyte migration and increases apoptosis. **(A)** Podocyte migration was increases by Adriamycin compared to the control, as analyzed using the scratch wound assay. Scale bar: 100 μm. **(B)** Quantitative analysis of the migration rates in **(A)**. **p < 0.01 (*n* = 3). **(C)** Migratory ability of Adriamycin-induced podocytes was further confirmed using the Transwell assay. Scale bar: 50 μm. **(D)** Quantitative analysis of the migrated cells in **(C)**. **p < 0.01 (*n* = 3). **(E)** Apoptotic podocytes were determined using flow cytometry. **(F)** Relative apoptosis rate of podocytes following Adriamycin treatment. **p < 0.01 (*n* = 3). **(G)** Cell viability of podocytes following Adriamycin treatment. **p < 0.01 (*n* = 3).

To explore the potential contribution of Sema3A to podocytopathy, we established an *in vitro* podocytopathy model using Adriamycin to induce injury in cultured podocytes. Initially, a scratch wound assay measured the effect of Adriamycin on podocyte migration, revealing a significant increase in podocyte motility (Figures 2A,B). The migratory ability of Adriamycin-treated podocytes was further confirmed using a Transwell assay, supporting enhanced cell migration with Adriamycin treatment (Figures 2C,D). Subsequently, apoptotic rates were determined, indicating a significantly higher ratio of apoptotic cells in the Adriamycin-treated group than in the control (Figures 2E,F). Accordingly, the cell viability upon treatment decreased compared to that of the control (Figure 2G). These data demonstrate that while Adriamycin promotes the migration of podocytes, it also induces cell death in cultured podocytes through apoptosis.

### 3.3 Increased Sema3A and Desmin expression and reduced podocin content in Adriamycin-induced podocytes

**Figure 3:**
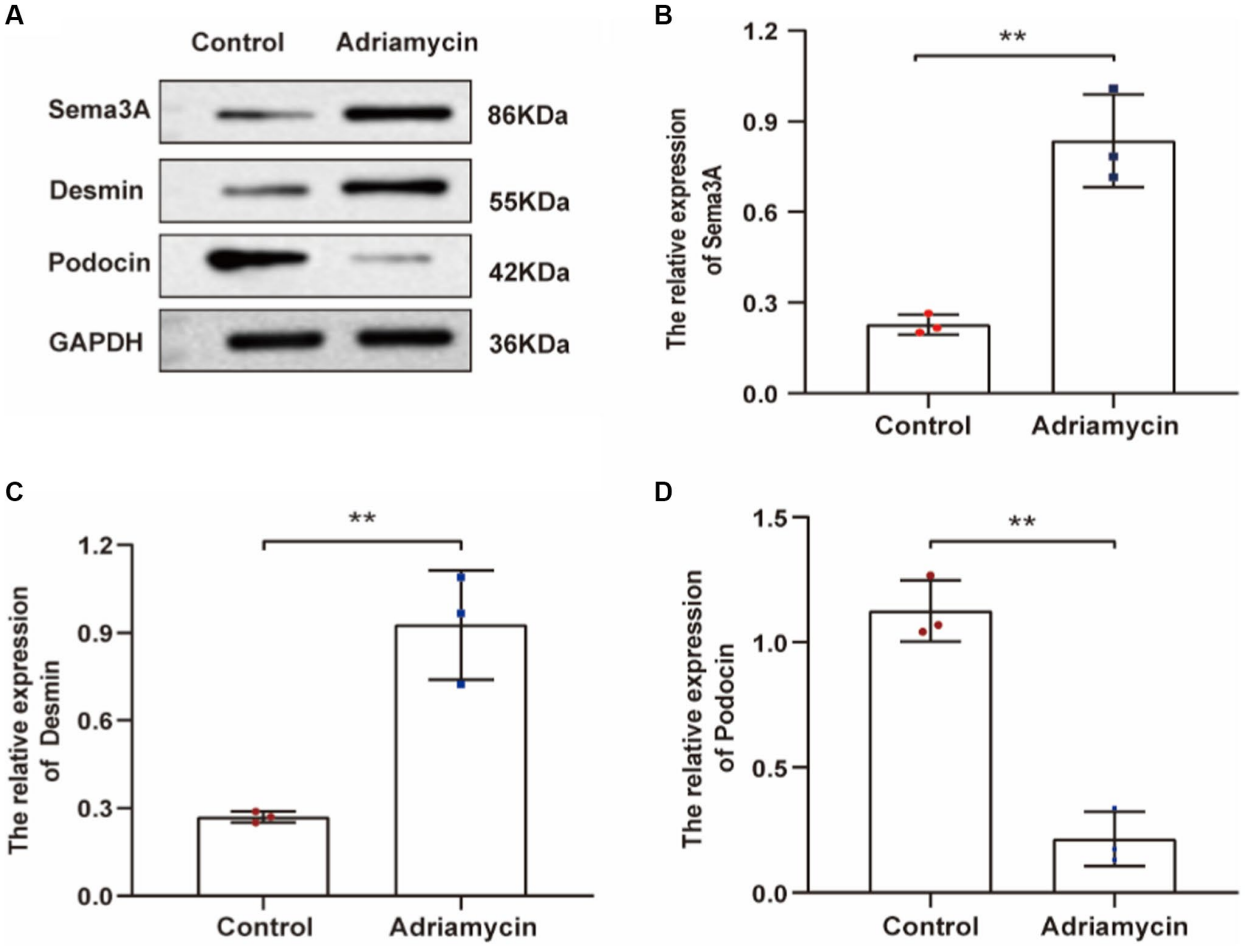
Adriamycin-induced protein expression in podocytes. **(A)** Western blot images of Sema3A, Desmin, and podocin protein expression in podocytes. **(B–D)** Relative quantitative analysis of Sema3A, Desmin, and podocin in Adriamycin-treated podocytes (*n* = 3). **p < 0.01.

Next, we examined the expression of Sema3A and the injury marker Desmin in Adriamycin-induced podocytes. The relative protein levels of Sema3A and Desmin significantly increased in Adriamycin-treated podocytes (*p* < 0.01) (Figures 3A–C), whereas the expression of the podocyte-specific protein podocin markedly decreased (*p* < 0.01) (Figures 3A,D). These findings demonstrate that Adriamycin upregulates Sema3A and Desmin expression while reducing podocin content in podocytes.

### 3.4 Inhibition of Sema3A protects podocytes from Adriamycin-induced injury

**Figure 4:**
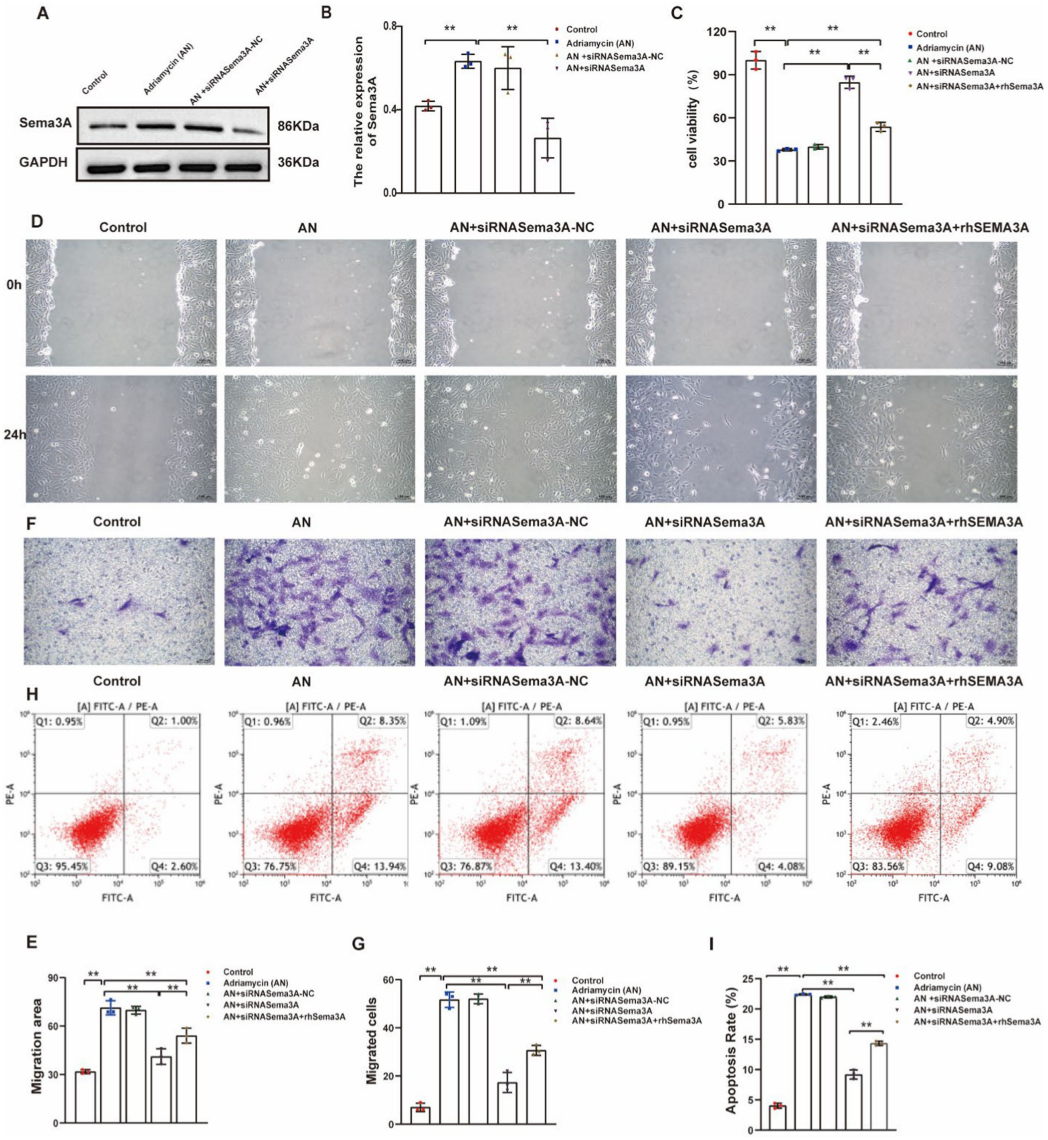
Inhibition of Sema3A protects podocytes from Adriamycin-induced injury. **(A)** Western blotting assay showing the levels of Sema3A in Adriamycin-induced podocytes. **(B)** Levels of Sema3A in Control, AN, Control siRNA (siRNASema3A-NC), and siRNASema3A, demonstrating a decrease in protein levels with siRNASema3A. **(C)** Cell viability of podocytes treated with AN+siRNASema3A and AN+siRNASema3A + rhSema3A (200 ng/mL in culture medium). **(D)** Podocyte migration area decreased in the siRNASema3A group compared with that in the AN and AN+siRNASema3A + rhSema3A groups, as analyzed using scratch wound assay. Scale bar: 100 μm. **(E)** Quantitative analysis of migration rates in **(D)**. **p < 0.01 (*n* = 3). **(F)** Podocyte migration decreased in the siRNASema3A group compared with that in the AN and AN+siRNASema3A + rhSema3A groups, as confirmed using the Transwell assay. Scale bar: 50 μm. **(G)** Quantitative analysis of migrated cells in **(E)**. **p < 0.01 (*n* = 3). **(H)** Determination of apoptotic podocytes using flow cytometry. **(I)** Relative apoptosis rate decreased following Adriamycin+siRNASema3A and Adriamycin +siRNASema3A + rhSema3A (200 ng/mL in culture medium) treatments compared with that after AN treatment (*n* = 3) **p < 0.01.

Our results demonstrate an increase in Sema3A expression in Adriamycin-induced podocytes. To verify the function of Sema3A in podocyte injury, we used siRNAs to inhibit the expression of Sema3A. Despite AN treatment enhancing the expression of Sema3A, the siRNA targeting Sema3A mRNA (siRNASema3A), not the scrambled control (siRNASema3A-NC), effectively reduced mRNA levels. Cellular Sema3A protein levels also showed a clear reduction after siRNA treatment (Figures 4A,B). Next, the CCK-8 assay was performed to analyze the impact of Sema3A on cell viability. The reduction of Sema3A by siRNA significantly reversed AN-induced cell death in podocytes. Re-expression of recombinant mouse Sema3A (rhSema3A), resistant to siRNASema3A, reproduced AN-induced cell death, indicating that AN-induced reduction of podocyte survival relies on the Sema3A protein (Figure 4C). Similarly, AN-induced enhancement of cell motility in podocytes depended on cellular Sema3A knockdown (AN+siRNASema3A) and re-expression (AN+siRNASema3A + rhSEMA3A), reducing and regaining AN-induced podocyte migration, respectively, as shown by the scratch wound assay (Figures 4D,E) and Transwell assay (Figures 4F,G). The ability of AN to induce podocyte apoptosis was weakened by Sema3A knockdown and restored by Sema3A overexpression, demonstrating that Sema3A is necessary for AN-induced podocyte death (Figures 4H,I). Overall, our data suggest that the effects of AN on podocytes are highly dependent on the cellular level of Sema3A, indicating that AN-induced podocyte injury is mediated by Sema3A.

### 3.5 Adriamycin-induced regulation of gene expression depends on Sema3A

**Figure 5:**
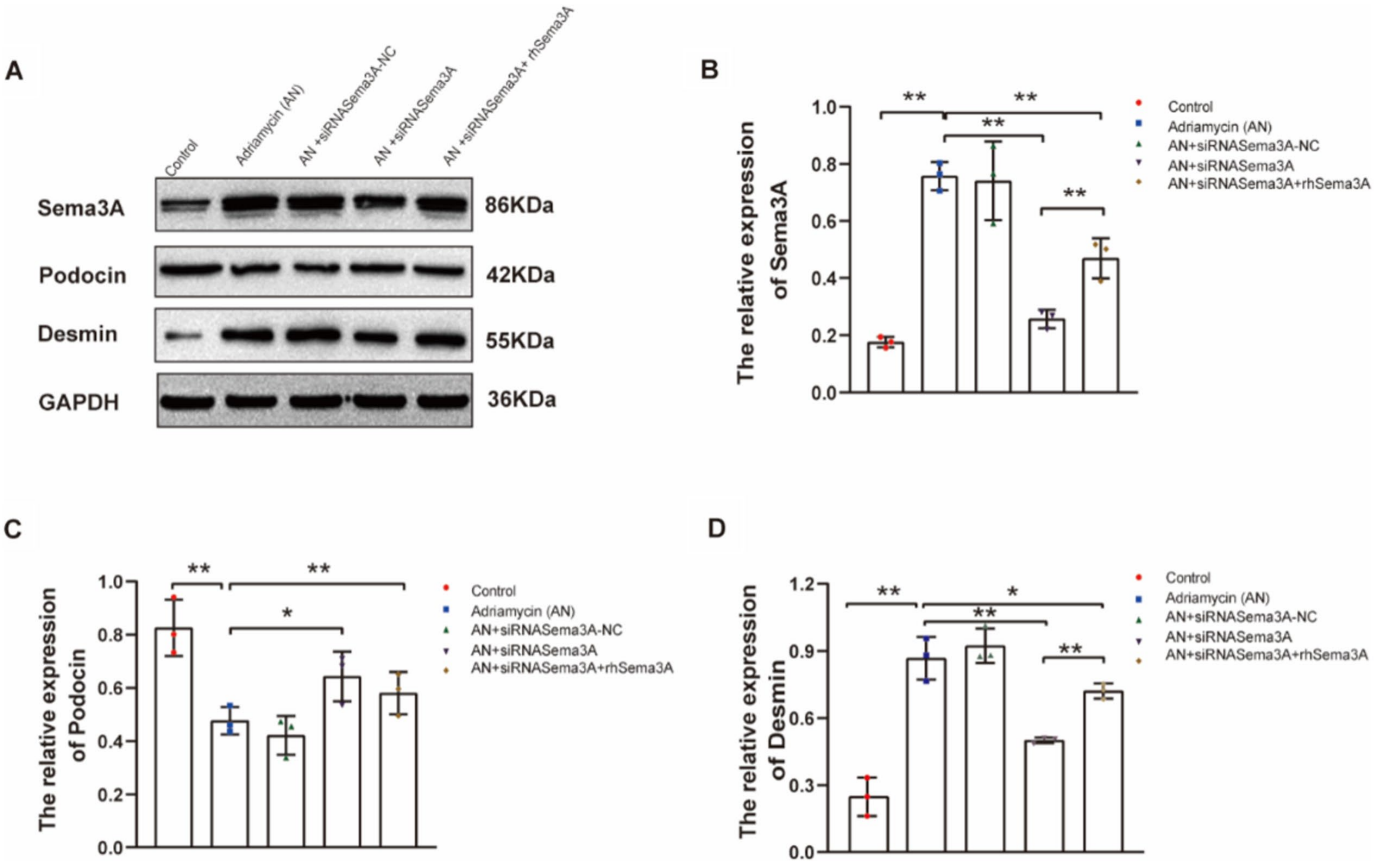
Inhibition of Sema3A increases the expression of podocin and decreases that of Desmin in podocytes. **(A)** Western blotting to assess the levels of Sema3A, podocin, and Desmin proteins in podocytes. **(B–D)** Relative densities of Sema3A, podocin, and Desmin protein expression normalized to GAPDH (*n* = 3) *p < 0.05, **p < 0.01.

We further investigated whether Sema3A is involved in regulating the expression of the injury marker Desmin and the podocyte marker podocin. The siRNA-mediated knockdown and exogenous re-expression of Sema3A were confirmed (Figures 5A,B). Adriamycin-induced podocin downregulation was counteracted by Sema3A knockdown (AN+siRNASema3A), whereas re-introducing Sema3A (AN+siRNASema3A + rhSema3A) restored the podocin loss caused by AN (Figures 5A,C). Although Desmin expression increased after AN treatment, in contrast to podocin, this upregulation was also dependent on Sema3A, the knockdown and re-introduction of which weakened and reproduced the Adriamycin-induced alteration in Desmin expression, respectively (Figures 5A,D). These data demonstrate that Sema3A is also indispensable for Adriamycin-induced regulation of podocin and Desmin expression.

**Figure 6:**
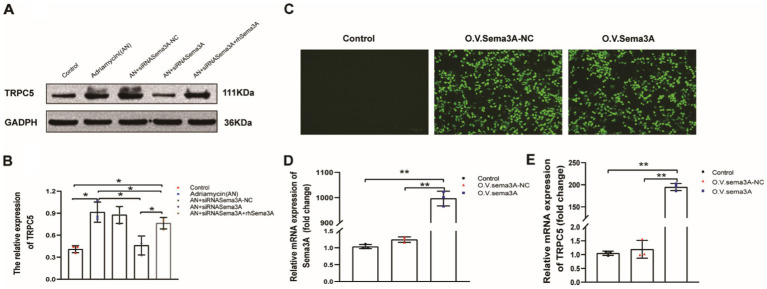
TRPC5 expression increased following Sema3A overexpression in podocytes. **(A)** Western blotting to assess TRPC5 levels in podocytes. **(B)** Upregulated TRPC5 expression in response to exogenous Sema3A (200 ng/mL) in the culture medium (*n* = 3) *p < 0.05. **(C)** Podocytes overexpressing Sema3A. **(D)** Relative mRNA expression of Sema3A and TRPC5 (*n* = 3) **p < 0.01. **(E)** Relative densities of Sema3A and TRPC5 protein expression normalized to GAPDH (*n* = 3) **p < 0.01.

We explored whether Sema3A regulates the expression of TRPC5, which is reported to be regulated by Sema3A in neurons, T cells, and the recombinant cytoskeleton. We examined the expression of TRPC5 in both enhanced cellular Sema3A and overexpressed Sema3A. TRPC5 expression in podocytes was dramatically increased (siRNASema3A + rhSema3A) while enhancing Sema3A (Figures 6A,B). In addition, we constructed an EGFP-N1 plasmid containing Sema3A cDNA to overexpress Sema3A in podocytes and found that the expression of TRPC5 was similar to that in Adriamycin-induced podocytes (Figures 6C–E). These results indicate that Sema3A induces podocyte injury via the Sema3A/TRPC5 signaling pathway.

### 3.6 TRPC5 inhibitors stabilize the cytoskeleton by inhibiting cell migration and alleviating cell injury induced by Adriamycin

**Figure 7:**
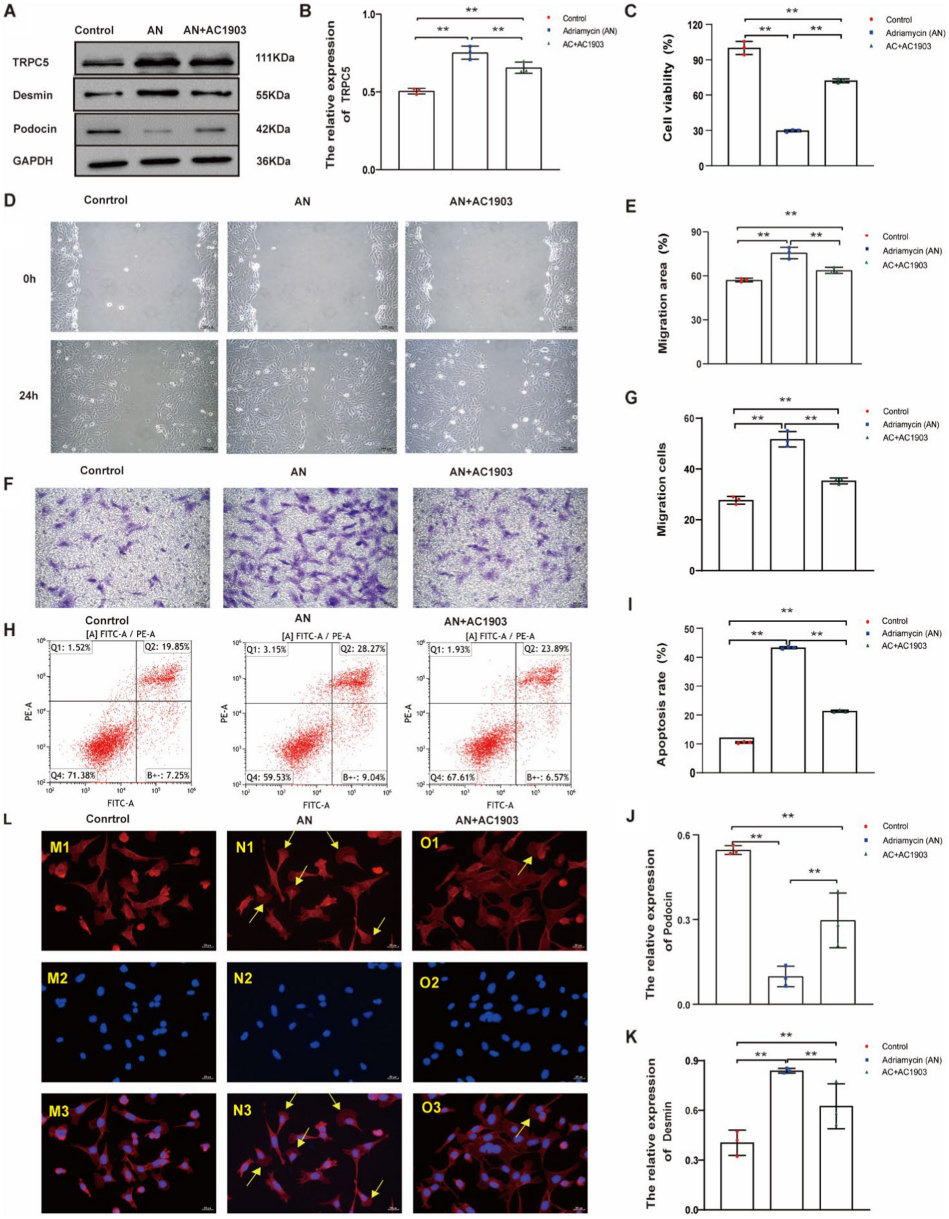
TRPC5 inhibitor alleviates Adriamycin-induced podocyte injury. **(A)** Western blotting to assess the levels of TRPC5, Desmin and podocin protein in podocytes. **(B)** TRPC5 level was significantly reduced in the AN+AC1903 group compared with that in the Control and AN group (*n* = 3) (**p < 0.01). **(C,D)** Relative densities of Desmin and podocin protein expression normalized to GAPDH (*n* = 3) **p < 0.01. **(E)** Podocyte migration area increased in the AN+AC1903 (4.06 μM) group compared with that in the AN group, as analyzed using a scratch wound assay. Scale bar: 100 μm. **(F)** Quantitative analysis of the migration rates in **(D)** (*n* = 3) **p < 0.01. **(G)** Podocyte migration increased in the AN+AC1903 (4.06 μM) group compared with that in the AN group, as confirmed using the Transwell assay. Scale bar: 50 μm. **(H)** Quantitative analysis of the migrated cells in **(F)** (*n* = 3) **p < 0.01. **(I)** Apoptotic podocytes were determined using flow cytometry. **(J)** Relative apoptosis rate of podocytes following AN and AN+AC1903 (4.06 μM) treatments (*n* = 3) **p < 0.01. **(K)** Cell viability rate of podocytes in the AN+AC1903 (4.06 μM) group was higher than that in the AN group (*n* = 3) (**p < 0.01). **(L)** The yellow arrow indicates the lamellipodium (scale bar = 20 μM). M1/N1/O1 represents three groups of podocyte actin cytoskeleton; M2/N2/O2 DAPI represents DAPI staining of the three groups of podocyte nuclei. M3/N3/O3 represents the merge of M1/M2, N1/N2, and O1/O2.

Next, we investigated whether the inhibition of TRPC5 expression could affect cell function and the cytoskeleton, thereby altering cell movement, using a small molecule, AC1903, which specifically inhibits TRPC5. The inhibitory effect of AC1903 was verified using western blotting (Figures 7A,B). To examine whether TRPC5 is involved in Adriamycin-induced podocyte injury, the protein was detected using western blotting. Notably, AC1903 significantly decreased the relative expression level of Desmin (Figures 7A,C) and increased that of podocin (Figures 7A,D) (**p < 0.01). The scratch wound and Transwell assays indicated that AC1903 markedly upregulated the migration of podocytes (Figures 7E–H). AC1903 also attenuated Adriamycin-induced apoptosis in podocytes (Figures 7I,J) (**p < 0.01). The cell viability rate was higher in the AN+AC1903 group than in the AN group (**p < 0.01) (Figure 7L). To examine whether TRPC5 is involved in the recombination of the cytoskeleton in Adriamycin-induced podocytes, we used laser confocal microscopy to observe changes in the cytoskeleton of podocytes by staining with the ghost cyclopeptide. The number of podocytes with typical lamellipodia was higher in this group than in other groups (Figure 7K). Overall, the *in vitro* functional assays of podocytes suggest that TRPC5 inhibitors stabilize the cytoskeleton by inhibiting cell migration and alleviating Adriamycin-induced cell injury.

### 3.7 Blocking the activation of Rac1 by inhibiting TRPC5 could prevent cell migration

**Figure 8:**
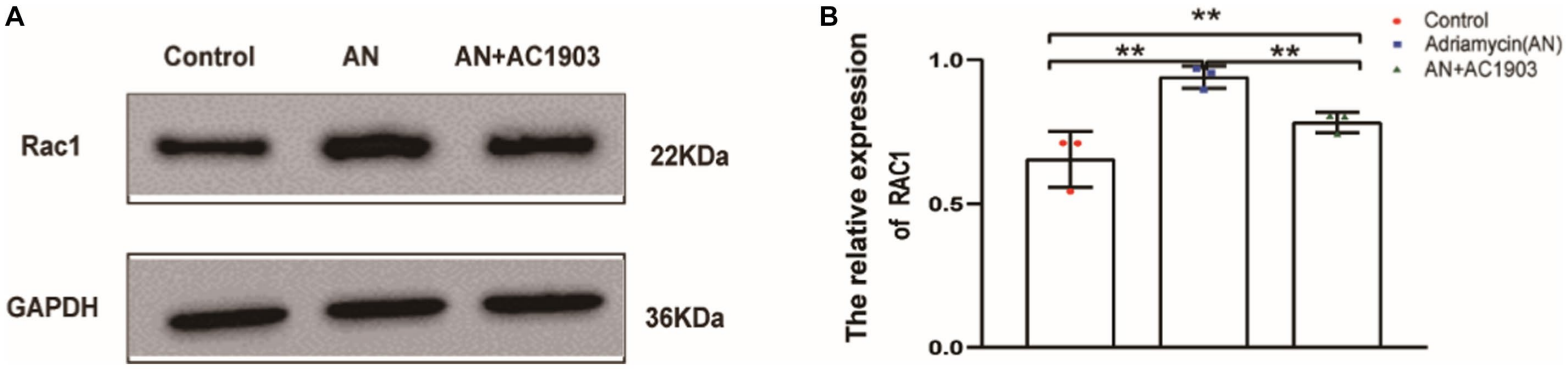
Inhibiting TRPC5 induce decrease Rac1 expression. **(A)** Western blotting to assess the levels of Rac1 protein in podocytes. **(B)** Relative densities of Rac1 protein expression normalized to GAPDH (*n* = 3) ^**^p < 0.01.

To confirm that TRPC5 participates in the reorganization of the cytoskeleton in podocytes, we detected Rac1 by inhibiting TRPC5. Rac1 activation promotes cytoskeletal restructuring, augmenting cell movement and migration. As evidenced by the western blotting images and quantitative data in Figures 8A,B, AC1903 decreased the protein levels of Rac1 in podocytes. These results suggest that inhibiting TRPC5 prevents cell migration by blocking Rac1 activation.

### 3.8 TRPC5 inhibitor alleviates 24-h urine protein and podocyte foot process fusion in the Adriamycin-induced mouse model

**Figure 9:**
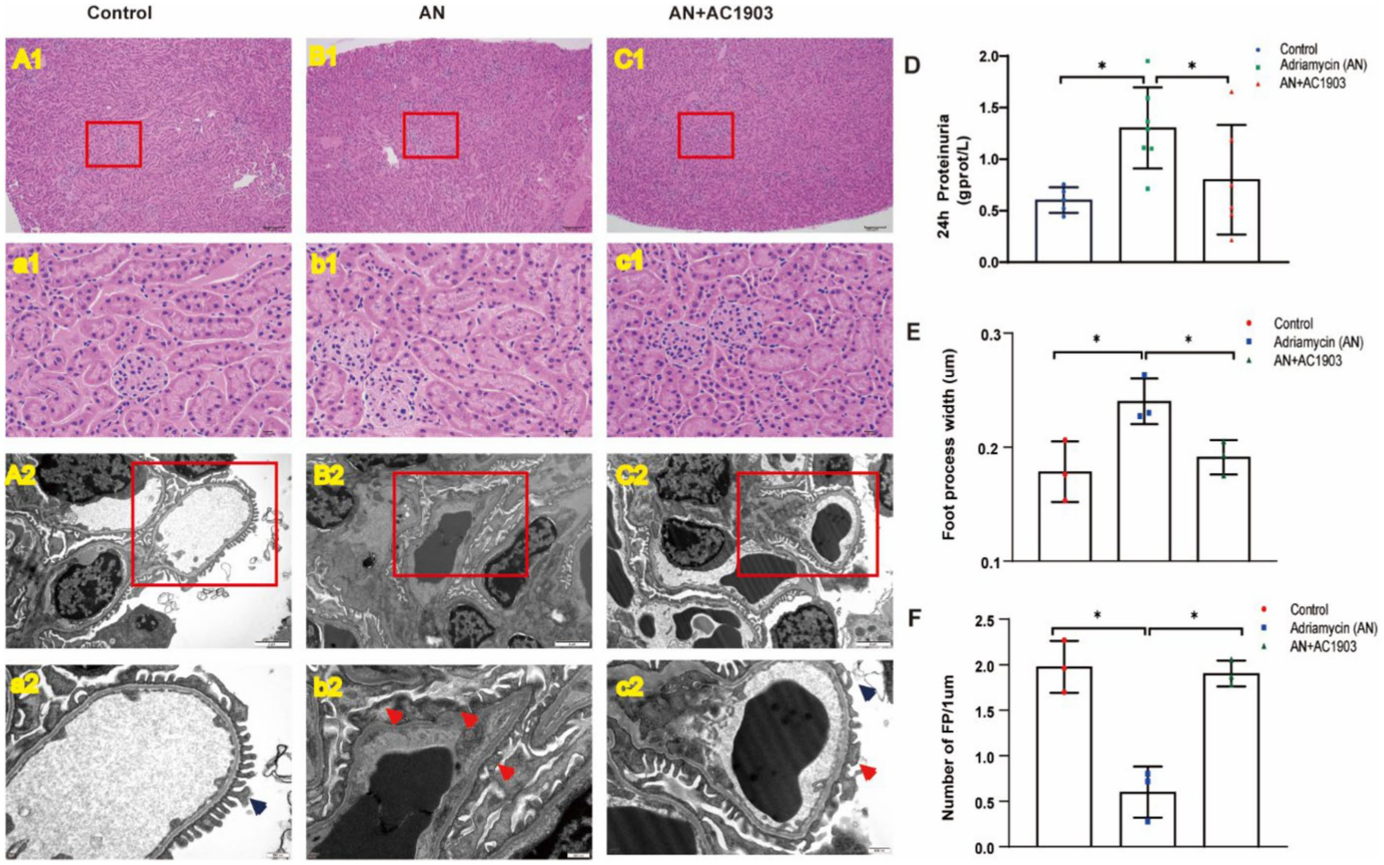
TRPC5 inhibitor protects podocyte in an Adriamycin-induced mouse model. **(A1/a1–C1/c1)** Histological manifestations were determined by HE staining to assess glomerular injury in the control, AN, and AN+AC1903 groups 11 days after AN injection. The kidney tissue in the red box of the **(A1–C1)** figure is zoomed, as shown in panels **(a1–c1)** (**A1–C1** bar = 100 μM, **a1–c1** bar = 10 μM). **(A2/a2–C2/c2)** Transmission electron microscopic image of the ultrastructure of the renal cortex in three groups of mice. The kidney tissue in the red box of the **(A2–C2)** figure is enlarged, as shown in panels **(a2–c2)** (**A2–C2** bar = 2 μM, **a2–c2** bar = 500 nm). The dark blue arrow shows the normal podocyte process, and the red arrow shows the fusion of the foot process. **(D)** Twenty-four-hour urinary protein at day 10 in three groups of mice ^*^p < 0.05. **(E,F)** Comparison of the number and width of foot processes in three groups of mice. **(E)** Comparison of the number of foot processes among the three groups (*n* = 3) ^*^p < 0.05. **(F)** Comparison of the width of foot processes in three groups of mice (*n* = 3) ^*^p < 0.05.

To explore the *in vivo* protective effect of the TRPC5 inhibitor on podocytes, we used an Adriamycin-induced podocytopathy mouse model. While HE staining showed no glomerular injury in the mouse kidneys after AN treatment (Figures 9A1/a1–C1/c1), transmission electron microscopy revealed extensive FPE in the AN group (Figures 9B2/b2). Continuous AC1903 administration for 10 days in the AN group preserved foot process integrity and fusion state (Figures 9C2/c2). In addition, we examined 24-h urinary protein levels, indicating that AN treatment alone resulted in the highest proteinuria among the three groups, and AC1903 reduced the urinary protein levels in the Adriamycin-induced mouse model (Figure 9D). Furthermore, the number and length of FPs were evaluated in the three groups, revealing a significantly higher number of FPs in the control and AN+AC1903 groups than in the AN-treated group. The length of FPs in the AN group was wider than that in the control and AN+AC1903 groups (Figures 9E,F). Collectively, TRPC5 inhibitors improved symptoms and restrained the fusion of foot processes.

### 3.9 TRPC5 inhibitor protects podocytes in Adriamycin-induced mouse model

**Figure 10:**
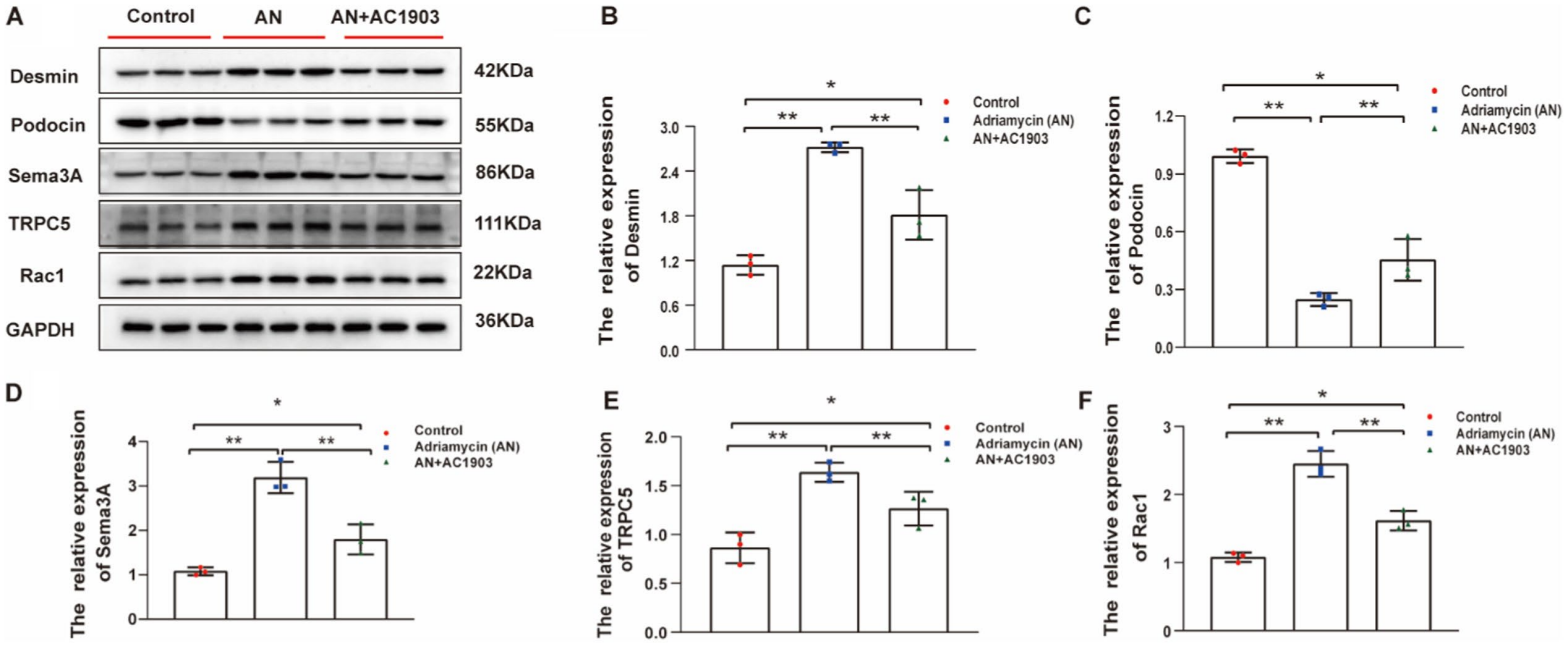
**(A)** Western blotting to assess the levels of Desmin, podocin Sema3A, TRPC5, and Rac1 protein in podocytes. **(B–F)** Relative protein expression of Desmin, podocin, Sema3A, TRPC5, and Rac1 in mice kidney tissue normalized to GAPDH were determined using western blotting. The values are presented as mean ± standard deviation (*n* = 3). ^*^p < 0.05, ^**^p < 0.01.

To substantiate the efficacy of the TRPC5 inhibitor *in vivo*, we examined the proteins that underwent alterations in the cell culture model of the Adriamycin-induced mouse model. Similarly, the expression levels of Desmin, Sema3A, podocin, TRPC5, and Rac1 showed significant variations in the mouse model. In Adriamycin-treated mice, Desmin, Sema3A, TRPC5, and Rac1 increased, whereas podocin decreased compared to that in the control. AC1903 treatment countered the effects of AN, leading to a substantial reversal of these changes (Figures 10A–F).

## 4 Discussion

In recent years, significant advances have occurred in our understanding of podocytes. Podocytes essentially float in the ultrafiltrate and attach to the glomerular basement membrane only through their processes. Podocytes transform into three distinct structural and functional elements: a large cell body, primary extension processes, and FPs (18). The distinctive interdigitating pattern of adjacent FPs creates snaking filter gaps between them, connected by an extracellular structure called a slit diaphragm (SD), which forms a physical barrier to the passage of large plasma proteins (19).

Proteinuria and nephrotic symptoms are the sole or primary clinical presentations of podocytopathies. MCD is one of the primary podocytopathies characterized by high proteinuria and nephrotic syndrome (20, 21). The lone morphologic feature identified by electron microscopy in renal biopsy shows “foot process effacement (FPE)” or flattening of podocyte foot processes. FPE indicates the loss of the usual interdigitating pattern of foot processes of adjacent podocytes, resulting in a relatively extensive expansion of podocyte processes covering the GBM (5). Foot process simplification and FPE represent the earliest morphological patterns of podocyte injury and can be associated with massive proteinuria even without podocyte loss. FEP is observed in numerous proteinuria kidney diseases (22). DKD represents secondary podocytes. Poorly controlled, long-standing diabetes mellitus can cause diabetic nephropathy with macroproteinuria as a manifestation of podocyte injury, which is the primary structural factor leading to decreased glomerular permeability in DKD (23). Podocyte loss, an irreversible event, is a key factor contributing to the progression of DKD and other podocytopathies (23–25). FP effacement in podocytes is remarkably dynamic and dependent on altered regulation of the podocyte cytoskeleton. The association between podocyte damage and albuminuria is well understood. Our results demonstrated that 24-h urinary proteinuria in patients with DKD, MN, MCD, and IgAN was higher than that in the control group; however, there was no difference in the various types of podocytopathies. FEP is not passive but rather a process highly based on signaling (26). It appears likely to be considered as the reestablishment of a more typical epithelial cell structure with broad and firm adhesion of the cells to their underlying basement membrane rather than pathological derangement (5). Thus, therapeutic targeting of podocytopathies may be a plausible avenue for exploration.

Sema3A is essential for the normal development of the glomerular filtration barrier and podocyte differentiation. A null mutation of Sema3A resulted in increased ureteric bud branching (27). Sema3A also plays a crucial role in podocyte differentiation and acts as an essential negative regulator of endothelial cell survival in developing glomeruli (15). Our study showed that urinary Sema3A levels were higher in patients with podocytopathy than in the controls, aligning with the findings of Inoue-Torii et al. (13) and Sang et al. (28). These results suggest the involvement of Sema3A in the pathological process of podocyte injury, prompting further examination of the possible mechanisms through cell and animal experiments.

Adriamycin is a commonly used drug for treating podocyte injury. *In vitro*, Adriamycin damages podocytes and disrupts the actin cytoskeleton (29). Our results showed that Adriamycin decreased podocyte proliferation and enhanced cell migration and podocyte apoptosis, consistent with previous studies.

Cell migration and shape change require actin cytoskeletal rearrangements. Podocytes regulate glomerular filtration barrier permeability via the recombination of the actin cytoskeleton. Sema3A is known for its ability to disrupt the actin cytoskeleton and disassemble F-actin into multiple cell types (30, 31). During T-cell activation, Sema3A blocks cytoskeletal reorganization in the DC/T-cell contact zone, thereby altering TCR polarization (30). Sema3A treatment increases the proportion of collapsed growth cones and regulates cytoskeletal dynamics in neurons (32, 33). Our results showed an increase in the expression of Sema3A and enhanced cell migration in Adriamycin-treated podocytes. Conversely, the blockade of Sema3A enhanced cell viability and inhibited cell migration and podocyte apoptosis. Exogenous Sema3A treatment replicated the results observed in Adriamycin-induced podocytes, indicating that Sema3A induces podocyte injury through cytoskeletal recombination.

The alteration of podocyte foot processes from a normal to effaced morphology requires the dynamic behavior of podocytes. During cell migration, a highly complex series of signals influences the regulation of the actin cytoskeleton. Glomerular podocytes express TRPC channels, a subclass of the larger TRP superfamily of Ca2+ permeable non-selective cation channels. TRPC5 has been shown to contribute to calcium influx in podocytes using electrophysiological and pharmacological methodologies (34). TRPC5 influences the permeability of the glomerular filtration barrier through its effects on the podocyte cytoskeleton (35). Our results showed a decrease in podocin, while TRPC5 and the podocyte injury marker protein Desmin increased in Adriamycin-induced podocytes. However, inhibiting the expression of Sema3A through siRNA in Adriamycin-induced podocytes resulted in increased podocin and decreased Desmin and TRPC5 protein levels. Exogenous Sema3A could restore this situation. Desmin expression aligns with the findings of Khalil et al. (36). Podocin, a critical component of the glomerular SD, plays a key role in maintaining SD integrity. Composed of 383 amino acids with a hairpin-like structure containing both cytoplasmic N-and C-terminal domains (37), podocin has also been implicated in human diseases. Podocin inactivation in mature kidneys causes focal segmental glomerulosclerosis and nephrotic syndrome (37). Mutations in podocin cause nephrotic syndrome (38, 39). In addition, TRPC5 was upregulated in Adriamycin-induced podocyte injury, and the overexpression of Sema3A in podocytes resulted in the downregulation of TRPC5 expression. *In vivo* results showed that TRPC5 inhibitors effectively alleviated the occurrence of Adriamycin-induced albuminuria in mice, consistent with the findings of Zhou et al. Morphologically, the foot processes were effaced in the kidney tissue of Adriamycin-treated mice, exhibiting wider podocyte width and fewer podocytes per unit length than in the control and TRPC5 inhibitor groups. This finding suggests that Sema3A may regulate the expression of TRPC5, contributing to cytoskeletal recombination in Adriamycin-induced podocyte injury.

Yu et al. demonstrated that regulators of the actin cytoskeleton, such as the small GTPase Rac1, lead to foot process effacement (40). Robins et al. found that enhanced Rac1 activation in podocytes caused podocyte detachment and glomerulosclerosis (41). The generation of calcium ion (Ca2+) microdomains is critical for promoting cell migration because they control the localized activity of Rho GTPases (35). Activation of Rac1 signaling leads to the vesicular insertion of TRPC5 ion channels into the podocyte plasma membrane, resulting in transient Ca2+ influx into the podocyte and further Rac1 activation. This forms a circuit that promotes podocyte cytoskeletal remodeling (42, 43). Our results confirmed that Adriamycin increased Rac1 expression in podocytes, whereas TRPC5 inhibitors decreased the expression of Rac1 both *in vitro* and *in vivo*. Rac1 regulates actin polymerization at the front to promote protrusion, and lamellipodia formation is dependent on Rac1 (44). Phalloidin staining of the actin cytoskeleton showed that lamellipodium was more evident in Adriamycin-induced podocytes than in the control and TRPC5 inhibitor groups.

We also pondered the discrepancy in the function of TRPC5 in the kidney compared to previous research. *In vivo* gain-in-function studies on TRPC5 with respect to renal function have yielded conflicting results. Wang et al. suggested that overexpression or activation of TRPC5 ion channels in C58BL/6 mice does not cause kidney barrier injury or aggravate albuminuria under pathological conditions. However, Zhou et al. showed that AC1903 inhibits progressive renal disease in AT1RTgSD rat models. This disparity may stem from variations in animal species and disease models used *in vivo*. C57BL/6 mice are more resistant to podocytotoxins, such as puromycin, amino-riboside, and Adriamycin-induced podocytotoxin damage, than BALB/c mice (45). The inbred mouse strains (C57BL/6) were more stable and repeatable than the non-inbred rat strains (Wistar or SD) (46). We used a small-molecule inhibitor of TRPC5 ion channels (AC1903) (17) to inhibit TRPC5 in mouse models to examine its therapeutic effect. Our results showed that AC1903 relieved 24-h urine protein and improved foot process fusion in an Adriamycin-induced BALB/c mouse model. Overall, we verified that Sema3A may induce cytoskeletal recombination of podocytes via TRPC5 ion channels, leading to FPE, and AC1903 could effectively reduce podocyte damage and contribute to cytoskeletal stabilization.

This study has some limitations, particularly in our inability to establish a clear relationship between Sema3A and TRPC5 *in vitro*. Two main factors contribute to this constraint. Firstly, the unavailability of commercial Sema3A inhibitors hinders our ability to conduct comprehensive *in vitro* investigations. Secondly, the utilization of Sema3A−/− mouse glomeruli as a model demonstrates wide foot processes and FPE (15) at birth, which does not align with the pathological process of FPE that can be reversed.

## 5 Conclusion

In summary, Sema3A overexpression induces podocyte injury, and the inhibition of Sema3A shows promise in alleviating such injury. Our findings suggest that Sema3A may influence the cytoskeletal stability of podocytes through TRPC5 ion channels via Rac1, resulting in foot process effacement. Moreover, AC1903 exhibits the potential to reverse Adriamycin-induced foot process fusion and urinary protein alterations. These insights provide a foundation for a potential therapeutic strategy aimed at preventing podocytopathies.

## Data Availability

The raw data supporting the conclusions of this article will be made available by the authors, without undue reservation.

## References

[1] Lv JC Zhang LX. Prevalence and disease burden of chronic kidney disease. Adv Exp Med Biol. (2019) 1165:3–15. doi: 10.1007/978-981-13-8871-2_1 31399958

[2] Liu BC Lan HY Lv LL. Renal Fibrosis: Mechanisms and Therapies: 1165. Singapore: Springer Singapore, (2019): 3–15. Available at: http://link.springer.com/10.1007/978-981-13-8871-2_1 (Accessed October 1, 2023).

[3] Schieppati A Remuzzi G. Chronic renal diseases as a public health problem: epidemiology, social, and economic implications. Kidney Int. (2005) 68:S7–S10. doi: 10.1111/j.1523-1755.2005.09801.x, PMID: 16108976

[4] Bommer J. Prevalence and socio-economic aspects of chronic kidney disease. Nephrol Dial Transplant. (2002) 17:8–12. doi: 10.1093/ndt/17.suppl_11.8, PMID: 12386249

[5] Kriz W Shirato I Nagata M LeHir M Lemley KV. The podocyte’s response to stress: the enigma of foot process effacement. Am J Physiol Renal Physiol. (2013) 304:F333–47. doi: 10.1152/ajprenal.00478.2012, PMID: 23235479

[6] Kopp JB Anders HJ Susztak K Podestà MA Remuzzi G Hildebrandt F. Podocytopathies. Nat Rev Dis Prim. (2020) 6:68. doi: 10.1038/s41572-020-0196-7, PMID: 32792490 PMC8162925

[7] Sarnak MJ. Implications of proteinuria: Ckd progression and cardiovascular outcomes. Adv Chronic Kidney Dis. (2011) 18:258–66. doi: 10.1053/j.ackd.2011.04.002 21782132

[8] Roth L Koncina E Satkauskas S Crémel G Aunis D Bagnard D. The many faces of semaphorins: from development to pathology. Cell Mol Life Sci. (2009) 66:649–66. doi: 10.1007/s00018-008-8518-z, PMID: 18953684 PMC11131483

[9] Reidy K Tufro A. Semaphorins in kidney development and disease: modulators of ureteric bud branching, vascular morphogenesis, and podocyte-endothelial crosstalk. Pediatr Nephrol. (2011) 26:1407–12. doi: 10.1007/s00467-011-1769-1 21336944 PMC3397149

[10] Xia J Worzfeld T. Semaphorins and Plexins in kidney disease. Nephron. (2016) 132:93–100. doi: 10.1159/000443645 26844545

[11] Ranganathan P Jayakumar C Mohamed R Weintraub NL Ramesh G. Semaphorin 3A inactivation suppresses ischemia-reperfusion-induced inflammation and acute kidney injury. Am J Physiol Renal Physiol. (2014) 307:F183–94. doi: 10.1152/ajprenal.00177.2014, PMID: 24829504 PMC4101625

[12] Jayakumar C Ranganathan P Devarajan P Krawczeski CD Looney S Ramesh G. Semaphorin 3A is a new early diagnostic biomarker of experimental and pediatric acute kidney injury. PLoS One. (2013) 8:e58446. doi: 10.1371/journal.pone.0058446, PMID: 23469280 PMC3587608

[13] Inoue-Torii A Kitamura S Wada J Tsuji K Makino H. The level of urinary semaphorin3A is associated with disease activity in patients with minimal change nephrotic syndrome. Int J Nephrol Renov Dis. (2017) 10:167–74. doi: 10.2147/Ijnrd.S132980, PMID: 28790860 PMC5489052

[14] Viazzi F Ramesh G Jayakumar C Leoncini G Garneri D Pontremoli R. Increased urine semaphorin-3A is associated with renal damage in hypertensive patients with chronic kidney disease: a nested case–control study. J Nephrol. (2015) 28:315–20. doi: 10.1007/s40620-014-0097-5, PMID: 24756974 PMC4207723

[15] Reidy KJ Villegas G Teichman J Veron D Shen W Jimenez J. Semaphorin3a regulates endothelial cell number and podocyte differentiation during glomerular development. Development. (2009) 136:3979–89. doi: 10.1242/dev.037267, PMID: 19906865 PMC2778745

[16] Tapia R Guan F Gershin I Teichman J Villegas G Tufro A. Semaphorin3a disrupts podocyte foot processes causing acute proteinuria. Kidney Int. (2008) 73:733–40. doi: 10.1038/sj.ki.5002726, PMID: 18075495

[17] Zhou Y Castonguay P Sidhom EH Clark AR Dvela-Levitt M Kim S. A small-molecule inhibitor of Trpc5 ion channels suppresses progressive kidney disease in animal models. Science. (2017) 358:1332–6. doi: 10.1126/science.aal4178, PMID: 29217578 PMC6014699

[18] Quaggin SE Kreidberg JA. Development of the renal glomerulus: good neighbors and good fences. Development. (2008) 135:609–20. doi: 10.1242/dev.001081 18184729

[19] Perico L Conti S Benigni A Remuzzi G. Podocyte–actin dynamics in health and disease. Nat Rev Nephrol. (2016) 12:692–710. doi: 10.1038/nrneph.2016.127, PMID: 27573725

[20] Vivarelli M Massella L Ruggiero B Emma F. Minimal change disease. Clin J Am Soc Nephrol. (2017) 12:332–45. doi: 10.2215/Cjn.05000516, PMID: 27940460 PMC5293332

[21] Chebotareva N Vinogradov A Mcdonnell V Zakharova NV Indeykina MI Moiseev S. Urinary protein and peptide markers in chronic kidney disease. Int J Mol Sci. (2021) 22:12123. doi: 10.3390/ijms222212123, PMID: 34830001 PMC8625140

[22] Reiser J Altintas MM. Podocytes [version 1; peer review: 2 approved]. F1000Research. (2016) 5:114. doi: 10.12688/f1000research.7255.1, PMID: 26918173 PMC4755401

[23] Weil EJ Lemley KV Mason CC Yee B Jones LI Blouch K. Podocyte detachment and reduced glomerular capillary endothelial fenestration promote kidney disease in type 2 diabetic nephropathy. Kidney Int. (2012) 82:1010–7. doi: 10.1038/ki.2012.234, PMID: 22718189 PMC3472108

[24] Kravets I Mallipattu SK. The role of podocytes and podocyte-associated biomarkers in diagnosis and treatment of diabetic kidney disease. J Endocrine Soc. (2020) 4:bvaa029. doi: 10.1210/jendso/bvaa029, PMID: 32232184 PMC7093089

[25] Ravaglia F Melica ME Angelotti ML de Chiara L Romagnani P Lasagni L. The pathology lesion patterns of Podocytopathies: how and why? Front Cell Dev Biol. (2022) 10:838272. doi: 10.3389/fcell.2022.838272, PMID: 35281116 PMC8907833

[26] Garg P. A review of podocyte biology. Am J Nephrol. (2018) 47:3–13. doi: 10.1159/000481633 29852492

[27] Tufro A Teichman J Woda C Villegas G. Semaphorin3a inhibits ureteric bud branching morphogenesis. Mech Dev. (2008) 125:558–68. doi: 10.1016/j.mod.2007.12.003 18249526 PMC3992265

[28] Sang Y Tsuji K Inoue-Torii A Fukushima K Kitamura S Wada J. Semaphorin3A-inhibitor ameliorates doxorubicin-induced podocyte injury. Int J Mol Sci. (2020) 21:4099. doi: 10.3390/ijms21114099, PMID: 32521824 PMC7312798

[29] Jeansson M Björck K Tenstad O Haraldsson B. Adriamycin alters glomerular endothelium to induce proteinuria. J Am Soc Nephrol. (2009) 20:114–22. doi: 10.1681/Asn.2007111205, PMID: 19073829 PMC2615716

[30] Lepelletier Y Moura IC Hadj-Slimane R Renand A Fiorentino S Baude C. Immunosuppressive role of semaphorin-3A on T cell proliferation is mediated by inhibition of actin cytoskeleton reorganization. Eur J Immunol. (2006) 36:1782–93. doi: 10.1002/eji.200535601, PMID: 16791896

[31] Dent EW. Netrin-1 and Semaphorin 3A promote or inhibit cortical axon branching, respectively, by reorganization of the cytoskeleton. J Neurosci. (2004) 24:3002–12. doi: 10.1523/Jneurosci.4963-03.2004, PMID: 15044539 PMC6729836

[32] Cai G Wu D Wang Z Xu Z Wong KB Ng CF. Collapsin response mediator protein-1 (Crmp1) acts as an invasion and metastasis suppressor of prostate cancer via its suppression of epithelial–mesenchymal transition and remodeling of actin cytoskeleton organization. Oncogene. (2017) 36:546–58. doi: 10.1038/onc.2016.227, PMID: 27321179 PMC5290039

[33] Kaczmarek JS Riccio A Clapham DE. Calpain cleaves and activates the Trpc5 channel to participate in semaphorin 3A-induced neuronal growth cone collapse. Proc Natl Acad Sci. (2012) 109:7888–92. doi: 10.1073/pnas.1205869109, PMID: 22547824 PMC3356680

[34] Ilatovskaya DV Staruschenko A. Trpc6 channel as an emerging determinant of the podocyte injury susceptibility in kidney diseases. Am J Physiol Renal Physiol. (2015) 309:F393–7. doi: 10.1152/ajprenal.00186.2015, PMID: 26084930 PMC4556891

[35] Schaldecker T Kim S Tarabanis C Tian D Hakroush S Castonguay P. Inhibition of the Trpc5 ion channel protects the kidney filter. J Clin Invest. (2013) 123:5298–309. doi: 10.1172/Jci71165, PMID: 24231357 PMC3859394

[36] Khalil SR Mohammed AT Abd El-Fattah AH Zaglool AW. Intermediate filament protein expression pattern and inflammatory response changes in kidneys of rats receiving doxorubicin chemotherapy and quercetin. Toxicol Lett. (2018) 288:89–98. doi: 10.1016/j.toxlet.2018.02.024 29474904

[37] Mollet G Ratelade J Boyer O Muda AO Morisset L Lavin TA. Podocin inactivation in mature kidneys causes focal segmental glomerulosclerosis and nephrotic syndrome. J Am Soc Nephrol. (2009) 20:2181–9. doi: 10.1681/Asn.2009040379, PMID: 19713307 PMC2754108

[38] Asharam K Bhimma R David VA Coovadia HM Qulu WP Naicker T. Nphs 2 V260E is a frequent cause of steroid-resistant nephrotic syndrome in black south African children. Kidney Int Rep. (2018) 3:1354–62. doi: 10.1016/j.ekir.2018.07.017, PMID: 30450462 PMC6224675

[39] Boute N Gribouval O Roselli S Benessy F Lee H Fuchshuber A. Nphs2, encoding the glomerular protein podocin, is mutated in autosomal recessive steroid-resistant nephrotic syndrome. Nat Genet. (2000) 24:349–54. doi: 10.1038/74166, PMID: 10742096

[40] Yu H Suleiman H Kim AHJ Miner JH Dani A Shaw AS. Rac1 activation in podocytes induces rapid foot process effacement and proteinuria. Mol Cell Biol. (2013) 33:4755–64. doi: 10.1128/Mcb.00730-13, PMID: 24061480 PMC3838009

[41] Zheng CJ Sohn MJ Kim WG. Vinaxanthone, a new FabI inhibitor from Penicillium sp. J Antimicrob Chemother. (2009) 63:949–53. doi: 10.1093/jac/dkp058, PMID: 19282328

[42] Wieder N Greka A. Calcium, Trpc channels, and regulation of the actin cytoskeleton in podocytes: towards a future of targeted therapies. Pediatr Nephrol. (2016) 31:1047–54. doi: 10.1007/s00467-015-3224-1 26490951 PMC4840088

[43] Tian D Jacobo SMP Billing D Rozkalne A Gage SD Anagnostou T. Antagonistic regulation of actin dynamics and cell motility by Trpc5 and Trpc6 channels. Sci Signal. (2010) 3:ra77. doi: 10.1126/scisignal.2001200, PMID: 20978238 PMC3071756

[44] Etienne-Manneville S Hall A. Rho Gtpases in cell biology. Nature. (2002) 420:629–35. doi: 10.1038/nature01148 12478284

[45] Lee VW Harris DC. Adriamycin nephropathy: a model of focal segmental glomerulosclerosis: Adriamycin nephropathy. Nephrology. (2011) 16:30–8. doi: 10.1111/j.1440-1797.2010.01383.x, PMID: 21175974

[46] Festing MFW. Inbred strains should replace outbred stocks in toxicology, safety testing, and drug development. Toxicol Pathol. (2010) 38:681–90. doi: 10.1177/0192623310373776, PMID: 20562325

