# Supplementary material for: Adriamycin-induced podocyte injury via the Sema3A/TRPC5/Rac1 pathway
Source: Front Med (Lausanne). 2024 Sep 5;11:1381479. doi: 10.3389/fmed.2024.1381479 (PMC11410697; doi:10.3389/fmed.2024.1381479)
Supplement: Supplementary file 1 [file Table_1.DOCX]

Supplementary Material

# Supplementary Figures and Tables

Supplementary Table 1 characteristics of the podocytopathies patients and controls

| Groups | n | male（%） | age(years) | BMI (kg/m^2^) |
| --- | --- | --- | --- | --- |
| IgAN | 17 | 8（47.06） | 36.29±13.50 | 24.88±5.12 |
| MN | 22 | 13（59.09） | 50.09±11.64^a^ | 24.64±3.29 |
| DKD | 68 | 35（51.47） | 57.69±12.14^ab^ | 25.21±3.60 |
| MCD | 7 | 3（42.86） | 51.71±17.62 ^ab^ | 23.57±3.21 |
| Controls | 21 | 11（52.38） | 53.05±10.17^a^ | 25.1±3.22 |
| *P* value |  | 0.931 | ＜0.001 | 0.826 |

Notes：Mean±SD are reported for the continuous variables. Percentages are reported for the categorical variables*,* ^a^ *P*＜0.01 versus IgAN，^b^ *P*＜0.01 versus MN*.*
